# 4-Fluoro­benzyl (*Z*)-2-(2-oxoindolin-3-yl­idene)hydrazine-1-carbodi­thio­ate

**DOI:** 10.1107/S2414314624002359

**Published:** 2024-03-19

**Authors:** Mohd Abdul Fatah Abdul Manan, David B. Cordes, Aidan P. McKay

**Affiliations:** a Faculty of Applied Sciences, Universiti Teknologi MARA, 40450 Shah Alam, Selangor, Malaysia; b EaStCHEM School of Chemistry, University of St Andrews, St Andrews, Fife KY16 9ST, United Kingdom; University of Aberdeen, United Kingdom

**Keywords:** crystal structure, di­thio­carbazate, fluorine, isatin, *z* configuration, hydrogen bond

## Abstract

The title compound, C_16_H_12_FN_3_OS, a fluorinated di­thio­carbazate imine derivative, was synthesized by the one-pot, multi-component condensation reaction of hydrazine hydrate, carbon di­sulfide, 4-fluoro­benzyl chloride and isatin. The compound demonstrates near-planarity across much of the mol­ecule in the solid state and a *Z* configuration for the azomethine C=N bond. The *Z* form is further stabilized by the presence of an intra­molecular N—H⋯O hydrogen bond. In the extended structure, mol­ecules are linked into dimers by N—H⋯O hydrogen bonds and further connected into chains along either [2  0] or [100] by weak C—H⋯S and C—H⋯F hydrogen bonds, which further link into corrugated sheets and in combination form the overall three-dimensional network.

## Abstract 2

The crystal structure of a new fluorinated di­thio­carbazate imine containing the isatin moiety, 4-fluoro­benzyl (*Z*)-2-(2-oxoindolin-3-yl­idene)hydrazine-1-carbodi­thio­ate, is described.

## Graphical abstract

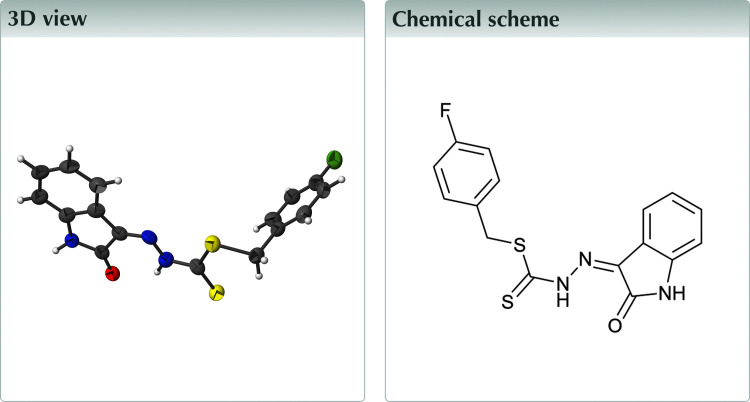

## Structure description

Various sulfur-containing mol­ecules isolated from natural sources have been reported to exhibit a broad spectrum of biological activities (Wang *et al.*, 2020 ▸; Chen & Li, 2023 ▸). Some synthetic sulfur-containing drugs inspired by natural products include the anti­biotics dalfopristin and quinupristin, and the anti­cancer agents phthalascidin and ixabepilone (Mustafa & Winum, 2022 ▸; Hai *et al.*, 2021 ▸). The ubiquitous role of fluorine in the design of bioactive mol­ecules is expending rapidly, as a better understanding of the unique properties of this element is gained. The introduction of a fluorinated substituent atom can influence p*K*_a_, basicity, dipole moment, conformation, intrinsic potency, membrane permeability, metabolic stability and pharmacokinetic properties (Richardson, 2021 ▸; Ali & Zhou, 2023 ▸). The literature reveals that various fluorine- and sulfur-containing drugs have been approved by the US Food and Drug Administration to combat diseases. Some examples are the recently reported lenacapavir for the treatment of HIV-1 infection (Paik, 2022 ▸; Han & Lu, 2023 ▸) and belzutifan for the treatment of kidney cancer (Deeks, 2021 ▸; Fallah *et al.*, 2022 ▸). As part of our ongoing studies in this area, we now describe the synthesis and structure of the title compound.

**Figure 1:**
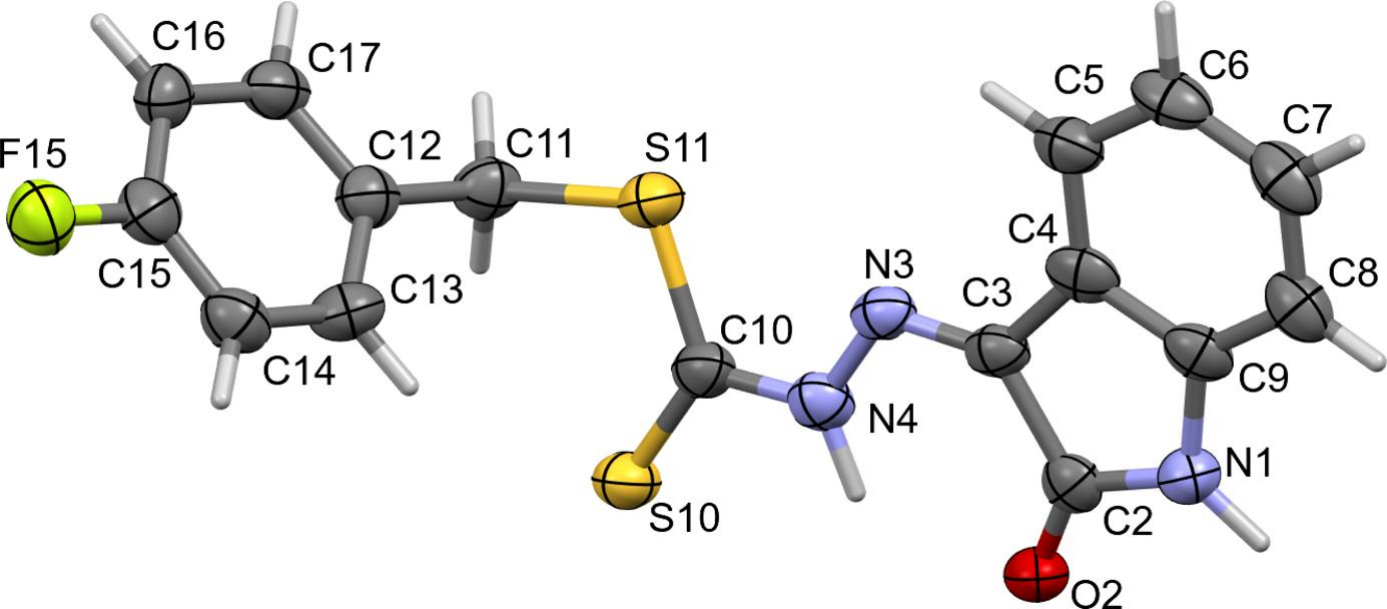
The mol­ecular structure of the title compound, showing displacement ellipsoids drawn at the 50% probability level.

The title compound crystallizes in the triclinic space group *P* 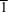 with one mol­ecule in asymmetric unit (Fig. 1 ▸). Its conformation and geometric details are similar to those in three closely related compounds; namely (*Z*)-benzyl 2-(5-methyl-2-oxoindolin-3-yl­idene)hydrazinecarbodi­thio­ate, benzyl 2-(5-chloro-2-oxo-1,2-di­hydro-3*H*-indol-3-yl­idene)hydrazine­carbo­di­thio­ate and benzyl 2-(5-bromo-2-oxo-1,2-di­hydro-3*H*-indol-3-yldene)hydrazinecarbodi­thio­ate (Abdul Manan *et al.*, 2011 ▸, 2023 ▸), the main difference being the dihedral angles between the aromatic rings and isatin moieties; 70.9° in the first, 72.6° in the second and 74.5° in the third compound, while in the title compound this dihedral angle is 82.6 (4)°.

**Figure 2:**
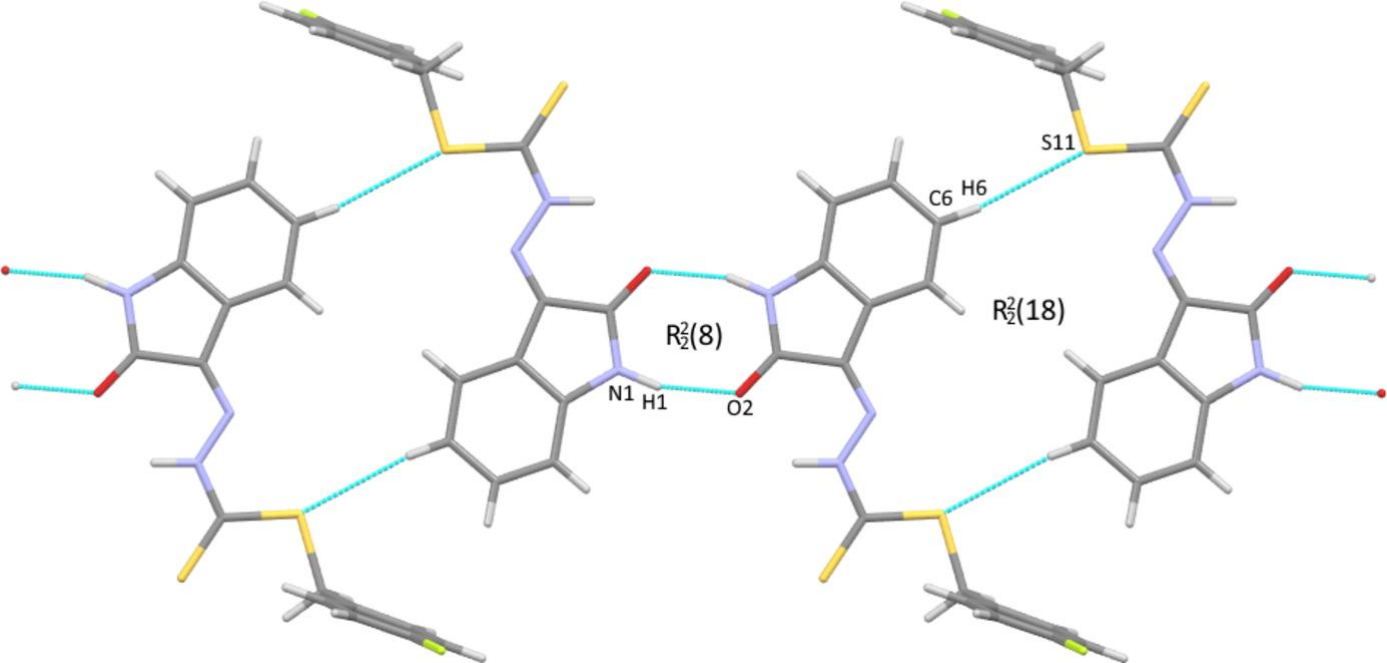
View of the hydrogen-bonded chains along [2 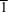 0] (left to right) formed from alternating N—H⋯O and C—H⋯S hydrogen-bonded dimers with 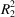 (8) and 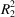 (18) motifs, respectively.

**Figure 3:**
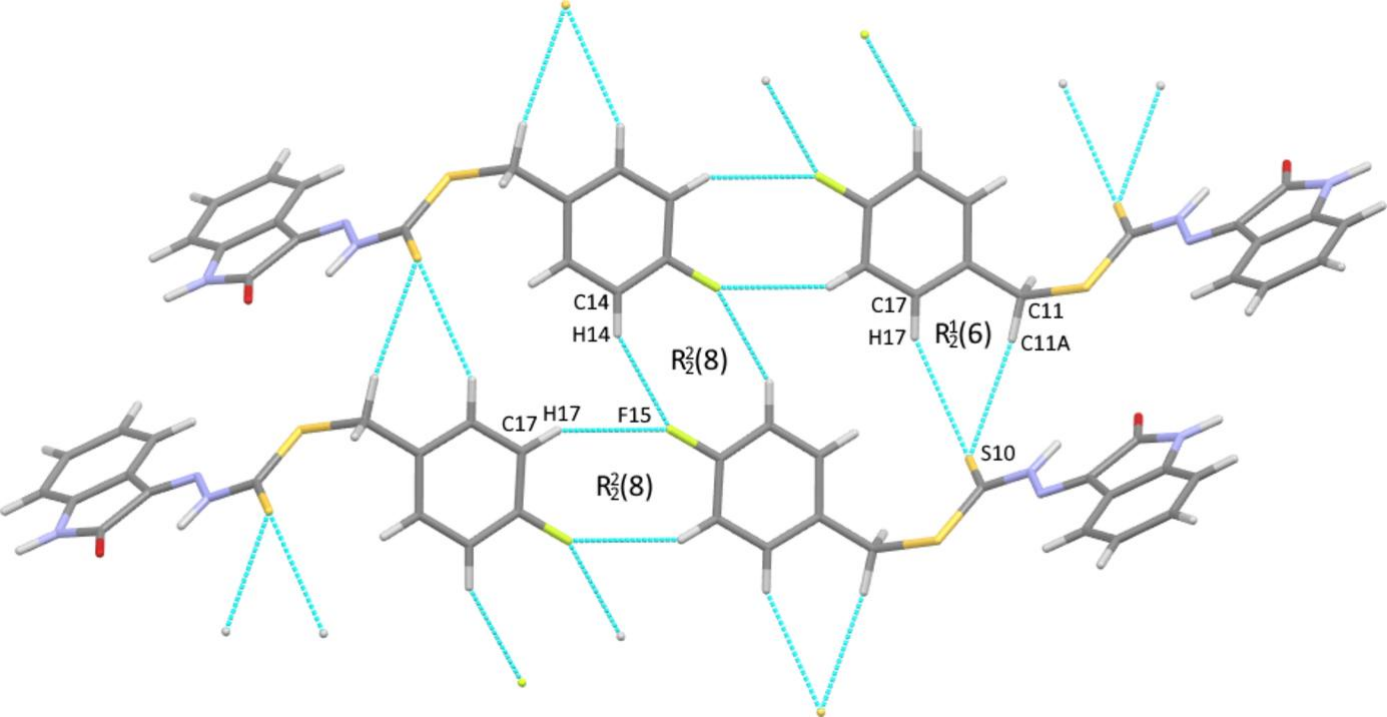
View of the hydrogen-bonded chains along [100] (top to bottom) formed from a combination of weak C—H⋯S and C—H⋯F hydrogen bonds with 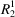 (6) and 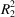 (8) motifs, respectively.

In the crystal of the title compound, individual mol­ecules form inversion dimers through pairwise N1—H1⋯O2 [H⋯O = 1.93 (6) Å, N⋯O = 2.844 (10) Å] hydrogen bonds (Table 1 ▸) in the common 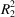 (8) motif. A second set of dimers is formed through weak C6—H6⋯S11 [H⋯S = 2.944 (3) Å, C⋯S = 3.819 (11) Å] hydrogen bonds in an 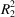 (18) motif, and the combination of the two dimeric inter­actions forms chains propagating along [2 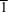 0] (Fig. 2 ▸). A second set of chains is formed by two pairs of weak hydrogen bonds: two donors, C11—H11*A* and C17—H17, inter­act simultaneously with S10 [H⋯S 2.918 (2) and 3.028 (3) Å, C⋯S = 3.876 (10) and 3.908 (11) Å] and the donors C14—H14 and C16—H16 inter­act in an alternating fashion with F15 [H⋯F = 2.523 (6) and 2.685 (7) Å, C⋯F = 3.347 (11) and 3.540 (12) Å], forming 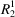 (6) and 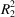 (8) motifs, respectively. This results in flat, tape-like chains running along [100] (Fig. 3 ▸), which can combine with either the N—H⋯O hydrogen-bonded dimers, or the weakly hydrogen-bonded dimer, giving corrugated sheets in both cases, lying in the (0 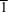 2) or (0 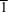 1) planes, respectively. The combination of these weaker inter­actions forms the overall three-dimensional structure.

## Synthesis and crystallization

30 ml of an ethano­lic solution of KOH (1.68 g, 0.03 mol, 1.0 eq) was mixed with hydrazine hydrate (1.50 g, 0.03 mol, 99%, 1.0 eq) and stirred at 0°C. Carbon di­sulfide (2.28 g, 0.03 mol, 1.0 eq) followed by 4-fluoro­benzyl chloride (4.34 g, 0.03 mol, 1.0 eq) were added to the initial mixture with constant stirring. After 1 h, 40 ml of an ethano­lic solution of isatin (4.42 g, 0.03 mol, 1.0 eq) were added and the resulting mixture was heated under reflux for 3 h. A yellow solid product was formed, which was then filtered and dried over silica gel, yielding yellow crystals on recystallization from ethanol solution (yield: 8.1 g, 78%). m.p. 214–215°C; ^1^H (400 MHz, *d*_6_-DMSO) δ: (p.p.m.): 4.51 (*s*, 2H), 6.90 (*d*, *J* = 7.89 Hz, 1H) 7.03 (*t*, *J* = 7.21 Hz, 1H), 7.13 (*t*, *J* = 17.69 Hz, 2H), 7.36 (*td*, *J* = 14.25, 8.19 Hz, 1H), 7.44–7.49 (*m*, 3H), 11.32 (*s*, 1H), 13.92 (*s*, 1H); ^19^F{^1^H} (376 MHz, *d*_6_-DMSO) δ: (p.p.m): −114.82; HRMS *m*/*z* (ESI^+^), found: [*M* + H]^+^ 346.0480, C_16_H_12_FN_3_OS_2_ requires [*M* + H]^+^ 346.0484.

## Refinement

Crystal data, data collection and structure refinement details are summarized in Table 2 ▸. The structure was refined as a two component twin with component 2 rotated by −179.99° around [−0.00 − 0.00 1.00] (reciprocal) or [−0.31 0.02 0.95] (direct), and a refined twin fraction of 0.451 (3).

## Supplementary Material

Crystal structure: contains datablock(s) I. DOI: 10.1107/S2414314624002359/hb4466sup1.cif

Structure factors: contains datablock(s) I. DOI: 10.1107/S2414314624002359/hb4466Isup2.hkl

Supporting information file. DOI: 10.1107/S2414314624002359/hb4466Isup3.cml

CCDC reference: 2339543

Additional supporting information:  crystallographic information; 3D view; checkCIF report

## Figures and Tables

**Table 1:** Hydrogen-bond geometry (Å, °)

*D*—H⋯*A*	*D*—H	H⋯*A*	*D*⋯*A*	*D*—H⋯*A*
N1—H1⋯O2^i^	0.96 (3)	1.92 (5)	2.845 (10)	159 (9)
N4—H4⋯O2	0.96 (3)	2.02 (8)	2.725 (10)	128 (8)
C6—H6⋯S11^ii^	0.95	2.94	3.819 (11)	154
C11—H11*A*⋯S10^iii^	0.99	2.92	3.876 (10)	163
C14—H14⋯F15^iv^	0.95	2.52	3.347 (11)	145
C16—H16⋯F15^v^	0.95	2.68	3.540 (12)	150
C17—H17⋯S10^iii^	0.95	3.03	3.908 (11)	155

Symmetry codes: (i) 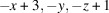 ; (ii) 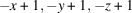 ; (iii) 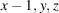 ; (iv) 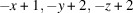 ; (v) 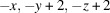 .

**Table 2:** Experimental details

Crystal data	
Chemical formula	C_16_H_12_FN_3_OS_2_
*M*_r_	345.41
Crystal system, space group	Triclinic, *P* 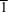
Temperature (K)	125
*a*, *b*, *c* (Å)	6.7949 (2), 6.9491 (2), 16.7080 (8)
α, β, γ (°)	89.525 (3), 82.547 (3), 82.347 (3)
*V* (Å^3^)	775.25 (5)
*Z*	2
Radiation type	Cu *K*α
μ (mm^−1^)	3.28
Crystal size (mm)	0.13 × 0.03 × 0.01

Data collection	
Diffractometer	Rigaku XtaLAB P200K
Absorption correction	Multi-scan (*CrysAlis PRO*; Rigaku OD, 2023 ▸)
*T*_min_, *T*_max_	0.651, 1.000
No. of measured, independent and observed [*I* > 2σ(*I*)] reflections	21967, 7828, 6291
*R*_int_	0.072
(sin θ/λ)_max_ (Å^−1^)	0.629

Refinement	
*R*[*F* ^2^ > 2σ(*F* ^2^)], *wR*(*F* ^2^), *S*	0.106, 0.268, 1.00
No. of reflections	7828
No. of parameters	215
No. of restraints	2
H-atom treatment	H-atom parameters constrained
Δρ_max_, Δρ_min_ (e Å^−3^)	0.89, −0.94

Computer programs: *CrysAlis PRO* (Rigaku OD, 2023 ▸), *SHELXT* (Sheldrick, 2015*a* ▸), *SHELXL2019/3* (Sheldrick, 2015*b* ▸), *Mercury* (Macrae *et al.*, 2020 ▸), *OLEX2* (Dolomanov *et al.*, 2009 ▸) and *publCIF* (Westrip, 2010 ▸).

## full crystallographic data

### Crystal data

**Table:**

C_16_H_12_FN_3_OS_2_	*Z* = 2
*M**_r_* = 345.41	*F*(000) = 356
Triclinic, *P*1	*D*_x_ = 1.480 Mg m^−^^3^
*a* = 6.7949 (2) Å	Cu *K*α radiation, λ = 1.54184 Å
*b* = 6.9491 (2) Å	Cell parameters from 4435 reflections
*c* = 16.7080 (8) Å	θ = 6.6–75.3°
α = 89.525 (3)°	µ = 3.28 mm^−^^1^
β = 82.547 (3)°	*T* = 125 K
γ = 82.347 (3)°	Needle, yellow
*V* = 775.25 (5) Å^3^	0.13 × 0.03 × 0.01 mm

### Data collection

**Table:**

Rigaku XtaLAB P200K diffractometer	7828 independent reflections
Radiation source: Rotating Anode, Rigaku MM-007HF	6291 reflections with *I* > 2σ(*I*)
Rigaku Osmic Confocal Optical System monochromator	*R*_int_ = 0.072
Detector resolution: 5.8140 pixels mm^-1^	θ_max_ = 76.0°, θ_min_ = 2.7°
shutterless scans	*h* = −8→8
Absorption correction: multi-scan (CrysAlisPro; Rigaku OD, 2023)	*k* = −8→8
*T*_min_ = 0.651, *T*_max_ = 1.000	*l* = −20→20
21967 measured reflections	

### Refinement

**Table:**

Refinement on *F*^2^	Primary atom site location: dual
Least-squares matrix: full	Hydrogen site location: mixed
*R*[*F*^2^ > 2σ(*F*^2^)] = 0.106	H-atom parameters constrained
*wR*(*F*^2^) = 0.268	*w* = 1/[σ^2^(*F*_o_^2^) + (0.020*P*)^2^ + 9.4*P*] where *P* = (*F*_o_^2^ + 2*F*_c_^2^)/3
*S* = 1.00	(Δ/σ)_max_ < 0.001
7828 reflections	Δρ_max_ = 0.89 e Å^−^^3^
215 parameters	Δρ_min_ = −0.94 e Å^−^^3^
2 restraints	

### Special details

**Table:**

Geometry. All esds (except the esd in the dihedral angle between two l.s. planes) are estimated using the full covariance matrix. The cell esds are taken into account individually in the estimation of esds in distances, angles and torsion angles; correlations between esds in cell parameters are only used when they are defined by crystal symmetry. An approximate (isotropic) treatment of cell esds is used for estimating esds involving l.s. planes.
Refinement. Refined as a 2-component twin.

### Fractional atomic coordinates and isotropic or equivalent isotropic displacement parameters (Å^2^)

**Table:**

	*x*	*y*	*z*	*U*_iso_*/*U*_eq_	
S10	0.9380 (4)	0.0909 (4)	0.82867 (14)	0.0439 (6)	
S11	0.5880 (4)	0.2351 (4)	0.73604 (14)	0.0429 (6)	
F15	0.2547 (9)	0.9804 (10)	0.9766 (3)	0.0562 (16)	
O2	1.3272 (9)	0.0761 (10)	0.5848 (4)	0.0400 (15)	
N1	1.2988 (12)	0.1631 (13)	0.4524 (5)	0.0385 (18)	
H1	1.430 (7)	0.105 (13)	0.430 (5)	0.046*	
N3	0.8885 (11)	0.2295 (11)	0.6073 (5)	0.0373 (18)	
N4	0.9629 (12)	0.1679 (13)	0.6748 (5)	0.0405 (19)	
H4	1.103 (6)	0.123 (14)	0.677 (6)	0.049*	
C2	1.2330 (14)	0.1453 (14)	0.5322 (6)	0.038 (2)	
C3	1.0120 (13)	0.2237 (14)	0.5419 (6)	0.037 (2)	
C4	0.9678 (15)	0.2821 (14)	0.4622 (6)	0.042 (2)	
C5	0.7917 (16)	0.3639 (15)	0.4326 (6)	0.045 (2)	
H5	0.670188	0.393144	0.467968	0.053*	
C6	0.7985 (17)	0.4011 (15)	0.3513 (6)	0.048 (3)	
H6	0.679127	0.452949	0.330486	0.058*	
C7	0.9757 (17)	0.3643 (15)	0.2992 (6)	0.049 (3)	
H7	0.976563	0.392205	0.243434	0.059*	
C8	1.1512 (17)	0.2875 (15)	0.3280 (6)	0.048 (3)	
H8	1.273295	0.263580	0.292639	0.057*	
C9	1.1449 (15)	0.2465 (15)	0.4086 (6)	0.041 (2)	
C10	0.8446 (14)	0.1609 (15)	0.7460 (6)	0.042 (2)	
C11	0.4666 (14)	0.2322 (15)	0.8402 (5)	0.043 (2)	
H11A	0.344241	0.168552	0.841732	0.051*	
H11B	0.558102	0.154445	0.873365	0.051*	
C12	0.4107 (14)	0.4338 (15)	0.8764 (5)	0.041 (2)	
C13	0.5556 (15)	0.5522 (16)	0.8848 (6)	0.046 (3)	
H13	0.692085	0.506535	0.866780	0.055*	
C14	0.5052 (16)	0.7368 (16)	0.9192 (6)	0.047 (2)	
H14	0.604631	0.817543	0.925452	0.056*	
C15	0.3072 (16)	0.7973 (17)	0.9436 (6)	0.047 (3)	
C16	0.1582 (15)	0.6873 (16)	0.9379 (5)	0.042 (2)	
H16	0.022436	0.734970	0.956436	0.051*	
C17	0.2117 (15)	0.5020 (17)	0.9037 (6)	0.046 (3)	
H17	0.111006	0.421622	0.899065	0.055*	

### Atomic displacement parameters (Å^2^)

**Table:**

	*U*^11^	*U*^22^	*U*^33^	*U*^12^	*U*^13^	*U*^23^
S10	0.0359 (12)	0.0552 (17)	0.0425 (12)	−0.0080 (12)	−0.0098 (10)	0.0027 (11)
S11	0.0303 (11)	0.0540 (16)	0.0450 (12)	−0.0058 (11)	−0.0070 (10)	−0.0004 (11)
F15	0.060 (4)	0.058 (4)	0.051 (3)	−0.015 (3)	−0.002 (3)	−0.008 (3)
O2	0.032 (3)	0.047 (4)	0.042 (3)	−0.003 (3)	−0.009 (3)	−0.002 (3)
N1	0.032 (4)	0.042 (5)	0.041 (4)	−0.004 (4)	−0.006 (3)	−0.001 (3)
N3	0.029 (4)	0.032 (5)	0.052 (4)	−0.005 (3)	−0.007 (3)	−0.005 (4)
N4	0.039 (5)	0.043 (5)	0.043 (4)	−0.014 (4)	−0.007 (3)	0.000 (4)
C2	0.040 (5)	0.030 (5)	0.045 (5)	−0.011 (4)	−0.007 (4)	−0.007 (4)
C3	0.032 (5)	0.032 (5)	0.049 (5)	−0.008 (4)	−0.010 (4)	−0.004 (4)
C4	0.045 (6)	0.032 (5)	0.054 (5)	−0.017 (5)	−0.016 (4)	−0.001 (4)
C5	0.043 (6)	0.044 (6)	0.050 (5)	−0.008 (5)	−0.017 (4)	−0.003 (5)
C6	0.056 (7)	0.038 (6)	0.056 (6)	−0.007 (5)	−0.030 (5)	0.007 (5)
C7	0.072 (8)	0.037 (6)	0.043 (5)	−0.009 (5)	−0.021 (5)	0.003 (4)
C8	0.062 (7)	0.041 (6)	0.044 (5)	−0.012 (5)	−0.014 (5)	−0.002 (4)
C9	0.045 (6)	0.037 (6)	0.046 (5)	−0.017 (5)	−0.014 (4)	0.000 (4)
C10	0.033 (5)	0.043 (6)	0.051 (5)	−0.007 (4)	−0.007 (4)	−0.009 (4)
C11	0.038 (5)	0.047 (6)	0.044 (5)	−0.013 (5)	−0.002 (4)	0.006 (4)
C12	0.040 (5)	0.047 (6)	0.035 (5)	−0.008 (5)	−0.005 (4)	0.005 (4)
C13	0.032 (5)	0.050 (7)	0.057 (6)	−0.005 (5)	−0.008 (4)	0.006 (5)
C14	0.050 (6)	0.046 (7)	0.047 (5)	−0.015 (5)	−0.010 (5)	0.001 (5)
C15	0.049 (6)	0.055 (7)	0.037 (5)	−0.006 (5)	−0.008 (4)	0.001 (5)
C16	0.041 (5)	0.051 (7)	0.034 (5)	−0.005 (5)	−0.001 (4)	0.000 (4)
C17	0.038 (5)	0.063 (8)	0.039 (5)	−0.015 (5)	−0.008 (4)	0.006 (5)

### Geometric parameters (Å, º)

**Table:**

S10—C10	1.637 (10)	C6—C7	1.387 (15)
S11—C10	1.780 (10)	C7—H7	0.9500
S11—C11	1.828 (9)	C7—C8	1.383 (14)
F15—C15	1.375 (12)	C8—H8	0.9500
O2—C2	1.213 (11)	C8—C9	1.371 (13)
N1—H1	0.96 (3)	C11—H11A	0.9900
N1—C2	1.360 (12)	C11—H11B	0.9900
N1—C9	1.412 (12)	C11—C12	1.512 (14)
N3—N4	1.339 (10)	C12—C13	1.386 (13)
N3—C3	1.286 (11)	C12—C17	1.391 (13)
N4—H4	0.96 (3)	C13—H13	0.9500
N4—C10	1.351 (12)	C13—C14	1.392 (15)
C2—C3	1.516 (13)	C14—H14	0.9500
C3—C4	1.445 (13)	C14—C15	1.365 (14)
C4—C5	1.403 (13)	C15—C16	1.360 (14)
C4—C9	1.399 (13)	C16—H16	0.9500
C5—H5	0.9500	C16—C17	1.396 (15)
C5—C6	1.376 (13)	C17—H17	0.9500
C6—H6	0.9500		
			
C10—S11—C11	102.7 (5)	C8—C9—N1	129.8 (10)
C2—N1—H1	121 (6)	C8—C9—C4	122.1 (10)
C2—N1—C9	112.3 (8)	S10—C10—S11	126.9 (6)
C9—N1—H1	126 (6)	N4—C10—S10	121.4 (7)
C3—N3—N4	117.2 (8)	N4—C10—S11	111.6 (7)
N3—N4—H4	124 (6)	S11—C11—H11A	109.1
N3—N4—C10	121.9 (8)	S11—C11—H11B	109.1
C10—N4—H4	114 (6)	H11A—C11—H11B	107.9
O2—C2—N1	128.1 (9)	C12—C11—S11	112.4 (7)
O2—C2—C3	126.4 (9)	C12—C11—H11A	109.1
N1—C2—C3	105.5 (8)	C12—C11—H11B	109.1
N3—C3—C2	126.7 (9)	C13—C12—C11	121.1 (9)
N3—C3—C4	127.3 (9)	C13—C12—C17	118.7 (10)
C4—C3—C2	105.9 (8)	C17—C12—C11	120.2 (9)
C5—C4—C3	132.9 (10)	C12—C13—H13	119.3
C9—C4—C3	108.3 (9)	C12—C13—C14	121.3 (10)
C9—C4—C5	118.9 (9)	C14—C13—H13	119.3
C4—C5—H5	120.7	C13—C14—H14	121.3
C6—C5—C4	118.7 (10)	C15—C14—C13	117.4 (10)
C6—C5—H5	120.7	C15—C14—H14	121.3
C5—C6—H6	119.3	C14—C15—F15	118.1 (9)
C5—C6—C7	121.4 (10)	C16—C15—F15	117.8 (9)
C7—C6—H6	119.3	C16—C15—C14	124.2 (11)
C6—C7—H7	119.8	C15—C16—H16	121.2
C8—C7—C6	120.4 (9)	C15—C16—C17	117.7 (10)
C8—C7—H7	119.8	C17—C16—H16	121.2
C7—C8—H8	120.8	C12—C17—C16	120.8 (10)
C9—C8—C7	118.5 (10)	C12—C17—H17	119.6
C9—C8—H8	120.8	C16—C17—H17	119.6
C4—C9—N1	108.1 (8)		
			
S11—C11—C12—C13	−61.5 (11)	C5—C4—C9—N1	−179.5 (8)
S11—C11—C12—C17	120.0 (8)	C5—C4—C9—C8	−0.6 (14)
F15—C15—C16—C17	179.4 (8)	C5—C6—C7—C8	0.5 (16)
O2—C2—C3—N3	−0.1 (15)	C6—C7—C8—C9	0.8 (15)
O2—C2—C3—C4	−177.7 (9)	C7—C8—C9—N1	177.9 (10)
N1—C2—C3—N3	177.8 (9)	C7—C8—C9—C4	−0.7 (15)
N1—C2—C3—C4	0.3 (10)	C9—N1—C2—O2	178.4 (9)
N3—N4—C10—S10	−178.3 (7)	C9—N1—C2—C3	0.5 (10)
N3—N4—C10—S11	1.7 (12)	C9—C4—C5—C6	1.8 (14)
N3—C3—C4—C5	2.4 (17)	C10—S11—C11—C12	103.8 (7)
N3—C3—C4—C9	−178.4 (9)	C11—S11—C10—S10	4.6 (8)
N4—N3—C3—C2	3.1 (13)	C11—S11—C10—N4	−175.4 (7)
N4—N3—C3—C4	−179.9 (9)	C11—C12—C13—C14	−179.0 (9)
C2—N1—C9—C4	−1.1 (11)	C11—C12—C17—C16	179.5 (9)
C2—N1—C9—C8	−179.9 (10)	C12—C13—C14—C15	−0.6 (15)
C2—C3—C4—C5	179.9 (10)	C13—C12—C17—C16	1.0 (14)
C2—C3—C4—C9	−0.9 (10)	C13—C14—C15—F15	−179.0 (9)
C3—N3—N4—C10	−177.4 (9)	C13—C14—C15—C16	1.4 (15)
C3—C4—C5—C6	−179.1 (10)	C14—C15—C16—C17	−0.9 (15)
C3—C4—C9—N1	1.2 (10)	C15—C16—C17—C12	−0.3 (14)
C3—C4—C9—C8	−179.9 (9)	C17—C12—C13—C14	−0.5 (15)
C4—C5—C6—C7	−1.8 (15)		

### Hydrogen-bond geometry (Å, º)

**Table:**

*D*—H···*A*	*D*—H	H···*A*	*D*···*A*	*D*—H···*A*
N1—H1···O2^i^	0.96 (3)	1.92 (5)	2.845 (10)	159 (9)
N4—H4···O2	0.96 (3)	2.02 (8)	2.725 (10)	128 (8)
C6—H6···S11^ii^	0.95	2.94	3.819 (11)	154
C11—H11*A*···S10^iii^	0.99	2.92	3.876 (10)	163
C14—H14···F15^iv^	0.95	2.52	3.347 (11)	145
C16—H16···F15^v^	0.95	2.68	3.540 (12)	150
C17—H17···S10^iii^	0.95	3.03	3.908 (11)	155

Symmetry codes: (i) −*x*+3, −*y*, −*z*+1; (ii) −*x*+1, −*y*+1, −*z*+1; (iii) *x*−1, *y*, *z*; (iv) −*x*+1, −*y*+2, −*z*+2; (v) −*x*, −*y*+2, −*z*+2.
